# Dike volume derived from seismicity as a gauge of fracture toughness and propagation dynamics

**DOI:** 10.1038/s41598-024-67724-0

**Published:** 2024-07-30

**Authors:** K. I. Konstantinou

**Affiliations:** Dept of Earth Sciences, National Central University, Jhongli, 320 Taiwan

**Keywords:** Dike, Eruption, Fracture toughness, Seismic moment, volcanic hazards, Seismicity, Natural hazards, Solid Earth sciences, Seismology, Volcanology

## Abstract

The temporal evolution of dike volume can help elucidate its propagation dynamics, however, such an estimation is possible only when there are geodetic observations available along the dike path. Here it is shown that dike volume history during eight eruptions can be reconstructed from seismic moment release using high resolution earthquake catalogs. The critical volume needed for each dike to reach the surface is simulated and compared to the accumulated volume prior to eruption in order to infer fracture toughness, a measure of resistance to fracture. It is found that fracture toughness varies between 123–833 MPa m ^1/2^, with larger values corresponding to longer dikes. Resistance to fracture dominates over viscous dissipation when the dikes propagate through unfractured heterogeneous material with large rigidity contrast, or when there is dike segmentation. These results can be utilized for real time monitoring of dike growth, forecasting eruption volume, and for constraining analog or numerical models of dike propagation.

## Introduction

Dikes are magma filled fractures that may reach the Earth’s surface producing an eruption, or alternatively may stall at some depth generating only deformation and seismicity. The importance of dikes in terms of magma transport and volcanic activity has prompted research on the mechanics of their propagation and the factors that influence their potential to feed an eruption^1–3^. On the basis of structural geology, seismic and geodetic observations as well as numerical modeling, there have been three main schools of thought about how dikes propagate towards the surface^4,5^. The first school considers that dikes are filled with melt of low viscosity and propagate towards the surface disconnected from the magma chamber, hence exhibiting a constant volume (‘Weertman model’). On the contrary, the second school of thought represents dikes as elongated features connected to the magma chamber, consisting of a long tail and a bulbous head filled with viscous melt (‘lubrication model’). Both of these representations assume that dikes always propagate in a direction close to the vertical, however, there is ample evidence (see Townsend et al.^5^) that under certain conditions dikes may switch their propagation direction from vertical to horizontal, as the third school of thought postulates. Several physical parameters can influence dike propagation, such as magma viscosity and its degree of bouyancy, as well as the mechanical properties of the host rock, all of which determine which of the aforementioned models explains better the observations.

A crucial parameter for the modeling of dike propagation is fracture toughness, which can be defined as the critical stress intensity factor that is necessary for a fracture to propagate^6^. Fracture toughness also influences the critical volume of dikes, that can be defined as the volume of fluid beyond which the dike will propagate in a self-sustained, uncontrollable manner to the surface^7^. There is considerable debate in the literature on whether fracture toughness can be considered as a material constant influenced by pressure and temperature, or whether its value depends primarily on the fracture length scale^8–11^. Measurements of fracture toughness obtained from small scale (${\sim }$60 mm) speciments of volcanic rocks^12^ exhibit a range between 1.4 and 3.8 MPa $\hbox {m}^{1/2}$ under variable temperature ($\le 750\,^{\circ }$C) and pressure ($\le$ 30 MPa). Such a limited range is in contrast to the reported values of fracture toughness estimated from the geometrical characteristics of solidified igneous dikes in Ethiopia^13^, Iceland^14^, and Japan^15^ that yielded a range of 38–273 MPa $\hbox {m}^{1/2}$. On the other hand, theoretical considerations^4^ and numerical modeling^16^ suggest that dikes should be associated with fracture toughness values in the order of hundreds of MPa $\hbox {m}^{1/2}$.

Despite the fact that fracture toughness is a well defined physical quantity adopted from material science, its amplitude and possible variation in volcanic environments remains poorly understood. In this context, there is a battery of hypotheses that can be formulated in order to highlight some of the yet unresolved issues. If fracture toughness is affected by material properties, then dike propagation in stratovolcanoes should be more influenced by resistance to fracture rather than viscous dissipation. This hypothesis is based on the fact that the edifice of stratovolcanoes consists of layers with different mechanical properties such as soft pyroclastic deposits and stiff lava flows, hence it requires large amounts of energy in order to fracture during intrusions^14^. Furthermore, if there is a dependency of fracture toughness on length scale, it would be expected that lateral dike intrusions, having lengths in the order of tens of kilometers, would exhibit larger fracture toughness than vertically propagating dikes whose length is usually smaller. It has also been suggested that after substantial cooling and solidification the length and thickness of a dike may be significantly different from those prior to these processes^17^. If this is the case, then the fracture toughness inferred from such static features will not be similar to that of dynamically propagating dikes where cooling only begins to take place. Unfortunately, to date these hypotheses cannot be tested due to the lack of fracture toughness estimates for dynamically propagating dikes.

**Figure 1:**
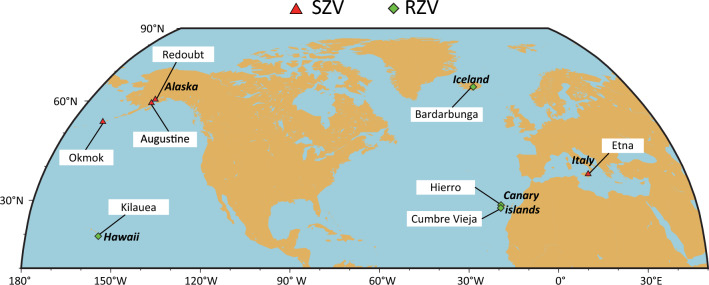
Map showing the locations of the eight volcanoes whose dike intrusions have been studied in this work (see text for more details). SZV: Subduction Zone Volcanoes, RSV: Rift Zone Volcanoes.

Dike volume can provide information on the level of fracture toughness and on the balance with viscous dissipation that ultimately determines the dynamics of dike propagation^4,18^. The volume history of propagating dikes is usually estimated from the modeling of deformation recorded by GPS receivers. One limitation regarding this approach has to do with the availability of such instruments only in well-monitored volcanoes. Another limitation is the fact that for dikes emanating from the top of magma chambers, the deformation due to the dike is usually overprinted by the simultaneous deflation of the magma chamber^19^. Recently it has been shown that intrusion volume can be derived from the cumulative seismic moment release of volcanotectonic earthquakes that often accompany dike propagation^20^. Here it is shown that a similar methodology can be utilized in order to reconstruct the volume history of dikes using high resolution earthquake catalogs from eight volcanoes situated in diverse volcanological settings (Fig. 1). The dike volume that accumulated prior to each eruption is compared to Monte Carlo simulations of critical volume in order to infer fracture toughness. The volume history of each dike is utilized for estimating the magma flux rate in order to investigate whether resistance to fracture or viscous dissipation dominates the propagation dynamics. Results show that dike volumes at the time of the eruption imply fracture toughness values that vary according to the dike length scale and are larger than those found for solidified dikes. Resistance to fracture becomes dominant over viscous dissipation when dikes propagate through previously unfractured, heterogeneous material or when there is dike segmentation.

## Results

### Seismic efficiency across volcanoes

For the purposes of this study eight well-monitored volcanoes have been selected that span different volcanological environments such as rift and subduction zones. Dike propagation in these volcanoes occurred in some cases laterally (Hierro, Bardarbunga, Kilauea), in other cases through the volcano edifice to the surface (Augustine, Etna, Okmok, Redoubt), or the dike followed a complicated path to the surface (Cumbre Vieja). The seismicity that accompanied these dike intrusions was recorded by local seismic networks whose data was processed for determining earthquake locations and magnitudes. In some volcanoes (Okmok, Bardarbunga, Kilauea, Cumbre Vieja) enhanced earthquake catalogs were produced by using waveform template matching or phase association with deep learning techniques that increased the quality and quantity of each catalog. This work makes use of these enhanced catalogs either on their own or by merging them with the routine catalogs provided by monitoring agencies (see Table 1 and the supplementary information). The analysis that will follow depends critically on the identification of a proper spatiotemporal window that will determine which events will be included. In terms of time, the window was set to start after the first signs of unrest in each volcano and to end when the eruption is over according to the global volcanism database of the Smithsonian Institution. The spatial extent of the window is determined by requiring that seismicity is located proximal to any resolved deformation sources, or by considering the seismicity closest to the plumbing system of the volcano. After this the completeness magnitude ($M_c$) was estimated for each of the eight catalogs. The method of *b*-value stability was utilized for the estimation of $M_c$, since it provides more precise estimates for volcanic earthquake catalogs^21,22^. The completeness magnitude was found in most cases to be smaller than 2.0 (Fig. S1 in suppl. information) precluding the possibility that larger events may have gone undetected. The supplementary information accompanying this work describes in more detail the available catalogs and how the spatiotemporal window was selected for each volcano.

A common link between volcanotectonic and induced earthquakes is that they both represent the response of the solid medium to fluid injection. In the case of induced earthquakes, McGarr^23,24^ showed that there is a relationship between the maximum earthquake magnitude expected after injection and the volume of the injected fluid. The relationship that connects the cumulative seismic moment release with volume change can be written as^20,25^

1 $$\begin{aligned} {\Delta }V S_{eff} G = \sum _{i=1}^{n} (M_{0})_{i} \end{aligned}$$

where ${\Delta }V$ is volume change, $(M_0)_{i}$ is the seismic moment for earthquake $i =$ 1, 2,...*n*, and *G* is the shear modulus. $S_{eff}$ is a quantity termed ‘seismic efficiency’ which represents the convolution of rock properties (lithological and petrophysical) with the earthquake productivity of the fluid intrusion. In order to utilize equation (1) all magnitudes were first converted to moment magnitude using appropriate empirical relationships, and then seismic moment was calculated by taking *G* equal to 3 GPa, which is close to the median value of shear modulus for volcanic rocks inferred from Monte Carlo simulations (see “Methods”). In this way ${\Sigma }M_0$ represents the cumulative seismic moment release until the end of the eruption, which should scale with estimates of the total erupted volume (see Table 1 and the supplementary information). Kettlety et al.^20^ used the same methodology with the difference that in their estimation of $S_{eff}$, intrusion volumes determined from geodetic observations were scaled with ${\Sigma }M_0$ released during each intrusion.

**Table 1:** List of eruptions and summary of parameters that were utilized in this study.

Eruption	V ($\textrm{m}^3$)	$\delta V (\textrm{m}^3)$	${\Sigma }\textrm{M}_0$ (Nm)	${\delta }{\Sigma }\textrm{M}_0$ (Nm)	$\textrm{S}_{eff}$	${\delta }\textrm{S}_{eff}$
Augustine (2006)	72.7e06	8.0e06	2.12e14	6.36e12	9.72e$-$04	1.10e$-$04
Etna (2008)	79.7e06	3.61e06	5.69e15	5.69e13	2.37e$-$02	1.10e$-$03
Okmok^*^ (2008)	0.21e09	0.05e09	2.48e15	4.96e13	3.93e$-$03	9.40e$-$04
Redoubt (2009)	10.0e07	5.0e07	6.73e14	2.01e13	2.24e$-$03	4.53e$-$04
Hierro (2011)	20.0e07	8.0e07	1.20e16	7.20e14	2.00e$-$02	8.08e$-$03
Bardarbunga^*^ (2014)	1.21e09	0.10e09	3.11e16	3.42e15	8.59e$-$03	1.18e$-$03
Kilauea^*^ (2018)	1.15e09	0.25e09	1.35e16	2.70e14	3.91e$-$03	8.54e$-$04
Cumbre Vieja^*^ (2021)	212.0e06	13.0e06	2.55e16	5.10e14	4.00e$-$02	2.58e$-$03

The column V refers to DRE volume (or bulk erupted volume for Etna and Cumbre Vieja) and ${\delta }$V is the corresponding unecrtainty (see the supplementary information for details). ${\Sigma }\hbox {M}_0$ is cumulative seismic moment until the end of each eruption and ${\delta }{\Sigma }\hbox {M}_0$ is its uncertainty. The last two columns represent the calculated seismic efficiency $\hbox {S}_{{eff}}$ and its uncertainty (${\delta }\hbox {S}_{{eff}}$). For eruptions indicated by an asterisk enhanced catalogs were utilized for the calculation of ${\Sigma }\hbox {M}_0$ and its uncertainty (see supplementary information).

**Figure 2:**
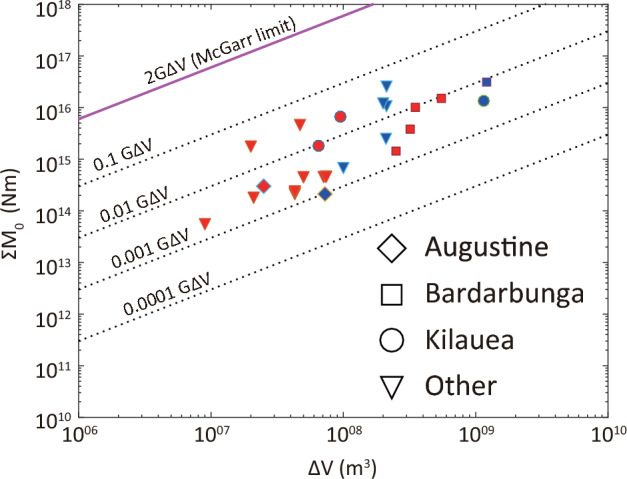
Diagram of volume change versus cumulative seismic moment release across different volcanoes. Red symbols represent data taken from Kettlety et al.^20^, adjusted for shear modulus $G =$ 3 GPa, where ${\Delta }$V represents intrusion volume determined geodetically. Blue symbols are the values derived for the eight volcanoes in this study where ${\Delta }$V represents total erupted volume (DRE or bulk). Dotted lines indicate isolines of constant seismic efficiency and the McGarr limit shows the theoretical maximum of seismic moment release. The three volcanoes plotted with distinct symbols are common between this work and Kettlety et al.^20^.

Figure 2 shows a plot of volume change versus the cumulative seismic moment for the datasets considered in this work as well as those of Kettlety et al^20^. In this plot the ‘McGarr limit’ represents the theoretical maximum value of cumulative seismic moment release during fluid injection. As shown by Kettlety et al.^20^, seismicity induced by human activities may reach, or in some cases, may even exceed this limit. On the contrary, seismicity caused by magmatic intrusions exhibits a far more limited range of moment release which results in a narrow range of values that $S_{eff}$ may take in volcanic environments. For all the volcanoes considered here seismic efficiency varies between $10^{-3}$ and $10^{-1}$ indicating that even though volcanoes cover different settings, their seismogenic properties appear to be similar. More specifically, for three volcanoes (Bardarbunga, Augustine, Kilauea) that are included both in this work and Kettlety et al.^20^, the different estimates of seismic efficiency vary by half an order of magnitude or less. This occurs despite the fact that ${\Delta }$V and ${\Sigma } \hbox {M}_0$ in each study correspond to very different time windows. The values of seismic efficiency along with their corresponding uncertainties (see Methods) are listed in Table 1. From this Table it can be inferred that the fractional uncertainty of seismic efficiency (${\delta }S_{eff}/S_{eff}$) is in most cases between 4 and 23%, except from Hierro that attains a value of 40%.

### Dike volume histories

The volume history of each dike can be reconstructed if the calculation of ${\Sigma }M_0$ is performed incrementally by adding one earthquake each time. The relationship that connects volume change with the cumulative seismic moment release as a function of time can be written as

2 $$\begin{aligned} {\Delta }V(t_{i}) = \frac{\sum _{i=1}^{n} M_{0}(t_{i})}{S_{eff} G} \end{aligned}$$

where the index $i =$ 1, 2,...*n* represents an event that occurred at time $t_{i}$, ${\Delta }V(t_{i})$ is volume change corresponding to that time, *G* is equal to 3 GPa, and seismic efficiencies correspond to the values determined earlier for each volcano (see Table 1). Fractional unecrtainty of seismic moment is calculated in the same way as in Kettlety et al.^20^ by summing under quadrature the fractional uncertainties of individual events (see Methods). As expected, the total uncertainty at the end of each earthquake sequence depends on the number of small magnitude (M < 2.0) events, since it is these that contribute the most to the uncertainty. In the eight earthquake catalogs that are considered here, the total fractional uncertainty is found to vary from a maximum of 11% for Bardarbunga down to 2–6% for all other volcanoes. The uncertainty of volume change can then be easily calculated from equation (2) by multiplying the fractional uncertainty of seismic moment with the corresponding value of ${\Sigma } \hbox {M}_0$ at each time $t_{i}$.

**Figure 3:**
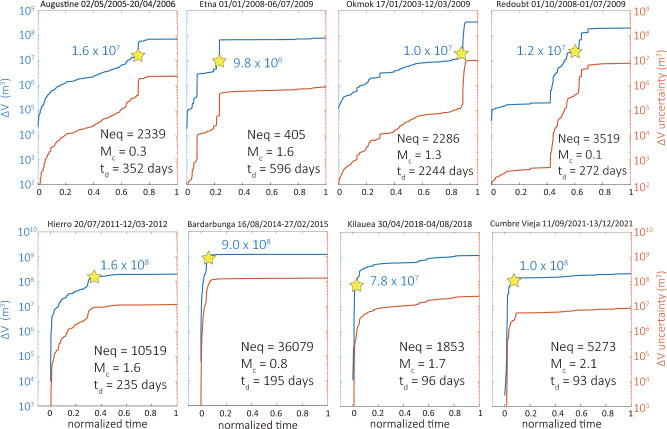
Dike volume histories and their uncertainties calculated for the eight dikes. The upper panel corresponds to subduction zone volcanoes and the lower one to rift zone volcanoes. At the top of each plot is the start and end date of the time window utilized. The yellow star denotes the time of the eruption and the number in blue fonts indicates the volume (in $\hbox {m}^3$) that accumulated in the dike up to that time. Neq is the total number of volcanotectonic earthquakes included in each catalog; $\hbox {M}_c$ is the magnitude of completeness; $\hbox {t}_d$ is the total duration of the time window which was used in order to normalize the time. Note that in the lower panels the origin of the x-axis has been slightly shifted to the right for clarity.

The reconstructed dike volume histories along with the evolution of their uncertainty are shown in Fig. 3, with the top panels corresponding to volcanoes along subduction zones and the lower ones to volcanoes situated in rift zones. A direct comparison of these volume histories would be difficult due to the fact that each catalog spanned different time length, therefore time was normalized by the total duration of each earthquake sequence. When visually compared, the dike volume histories of the two groups exhibit discernible differences that suggest diverse flow and propagation dynamics. The first of these differences is that ${\Delta }$V curves for volcanoes along subduction zones appear to be more jagged and volume change keeps accumulating even after the eruption has started. The curves of volcanoes in rift zones are smooth and do not vary significantly after the start of the eruption with the exception of Kilauea. A second difference is the fast dike inflation prior to eruption at volcanoes in rift zones, compared to the sluggish increase of volume at volcanoes in subduction zones with the exception of Etna. These two differences are likely related to the lower viscosity of basaltic magma filling the dikes in these volcanoes, where the fast flowing melt can inflate the dike rapidly. Another factor that may have played an important role is the occurrence (or not) of magma fragmentation in the dike. Numerical modeling has shown that with volatile exsolution, but no fragmentation at the dike tip, magma flux increases leading to an accelerated dike growth^26^. The opposite process occurs when fragmentation takes place, with the development of a large overpressure at the dike tip that results in deceleration of dike growth. In this context, dikes in volcanoes along subduction zones are more likely to experience fragmentation due to their volatile rich magma, which may explain the slow accumulation of volume change.

The dike volumes prior to eruptions can be compared to those obtained from modeling of geodetic observations in order to glean some insight into the variability of the different estimates. For three of the dikes studied here there are such estimates, namely for $\hbox {Bardarbunga}^{27}$ ${\sim }$51${\times } 10^{7} \hbox {m}^3$, Kilauea^28^ ${\sim }$10${\times } 10^{7} \hbox {m}^3$, and $\hbox {Okmok}^{29}$ ${\sim }$2.18${\times } 10^{7} \hbox {m}^3$. The percent difference between the two volume estimates is 81% for Bardarbunga, 21% and 53% for Kilauea and Okmok respectively (seismically derived volume being always smaller). The large difference in the two volume estimates for the Bardarbunga dike likely stems from the higher uncertainty in both seismic and geodetic observations. The former is related to uncertainty in the magnitude of small ($M_L<$ 2) earthquakes that make up the majority of the seismicity. The latter has to do with the limited coverage of the GPS receivers network within the Vatnajökull glacier where the largest part of the dike was situated^27^. The smaller seismic volume in Kilauea and Okmok may stem from aseismic deformation and also from the fact that deformation at Okmok started before the seismic network was installed^30^.

### Dike volume as a gauge of fracture toughness

According to linear elastic fracture mechanics a two-dimensional dike will always reach the Earth’s surface and cause an eruption, provided that there are no other factors that may affect adversely its propagation^4^. However, analog experiments that simulated dike propagation have revealed that even under optimal conditions three-dimensional dikes do not always reach the surface^1,31^. These observations have put forward the notion of critical volume, which is the volume of melt that should be exceeded in order for a dike to reach the surface and feed an eruption. Davis et al.^7^ performed numerical simulations of three-dimensional fractures filled with a variety of fluids (water, gas, magma) encased within rock of variable petrophysical properties. Based on the results of their simulations the authors proposed the following equation for the estimation of critical volume

3 $$\begin{aligned} V_c = \frac{1 - {\nu }}{16G} \Big (\frac{9 {\pi }^4 K_c^8}{{\Delta }{\gamma }^5 \cos ^5{{\theta }}}\Big )^{\frac{1}{3}} \end{aligned}$$

where ${\nu }$ is the Poisson ratio of the rock, $K_c$ is the fracture toughness, ${\theta }$ is the dike angle away from the vertical (thus for vertical dikes ${\theta } = 0^{\circ }$ and for horizontal ones ${\theta } = \textrm{90}^{\circ }$), ${\Delta }{\gamma }$ is equal to ${\Delta }{\rho }g =({\rho }_r - {\rho }_m)g$, with ${\rho }_r$ being the density of rock and ${\rho }_m$ the density of magma. Critical volume is proportional to $K_c^{2.6}$ which demonstrates that fracture toughness exerts the strongest influence on the value of $V_c$ relative to the other parameters.

**Figure 4:**
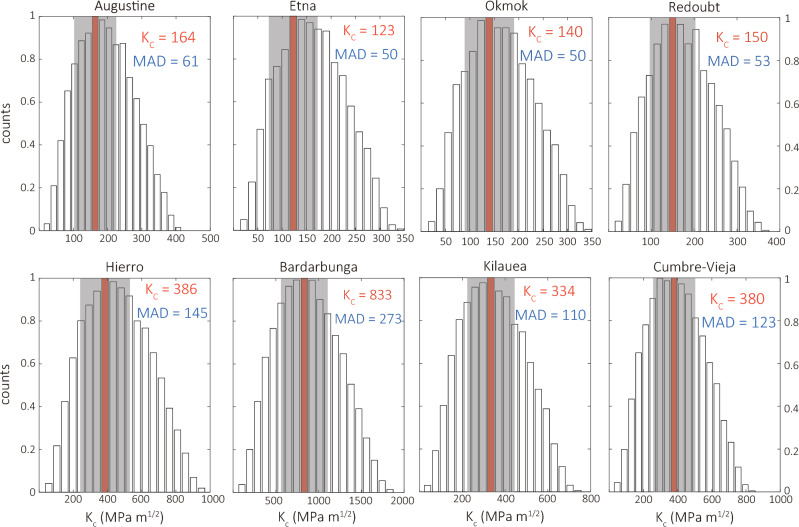
Normalized histograms of fracture toughness that correspond to dike volumes equal to 0.75–1.0 times the critical volume. The red bar highlights the mode of each distribution. The shaded area shows the extent of the Median Absolute Deviation (MAD) on either side of the mode. The values of the mode and MAD (in MPa $\hbox {m}^{1/2}$) for each distribution are included at the top right corner of each plot.

The numerical simulations indicated that a minimum volume equal to 0.75$V_c$ needs to accumulate in order for a dike to propagate to the surface. It is possible to infer fracture toughness by taking advantage of this relationship between $V_c$ and the dike volume that accumulated up to the occurrence of each eruption. Towards this end, a Monte Carlo simulation of $V_c$ is performed for 1.5 million times where all parameters ($K_c$, ${\theta }$, ${\Delta }{\rho }$, *G*, ${\nu }$) are allowed to vary randomly within physically realistic ranges (see Methods). Values of fracture toughness that correspond to $V_c$ between 0.75 and 1.0 times the dike volume prior to eruption are then selected and their distribution is shown in the form of normalized histograms in Fig. 4. In each histogram the mode of the distribution is highlighted along with the Median Absolute Deviation (MAD) defined as the median of $|K_c - \bar{K_c}|$, where $\bar{K_c}$ is the mean fracture toughness of each distribution. The value of the mode represents the most probable value of fracture toughness, while MAD is a statistically robust metric of variability that serves as a measure of uncertainty.

For the volcanoes along subduction zones the range of fracture toughness obtained in this way is 123–150 MPa $\hbox {m}^{1/2}$, while for those along rift zones the range becomes 334–386 MPa $\hbox {m}^{1/2}$. For the Bardarbunga dike the seismically derived volume suggests a fracture toughness of 833 MPa $\hbox {m}^{1/2}$, while for the geodetically derived volume this value is 683 MPa $\hbox {m}^{1/2}$. This implies a difference in $K_c$ of about 21% which is much smaller than the difference between the two volume estimates (81%). For the other two volcanoes where geodetic dike volumes are available this difference is 3% and 25% for Kilauea and Okmok respectively. Normalized histograms of fracture toughness inferred from geodetically determined volumes can be found in the supplementary information (Fig. S2). The robustness of the fracture toughness estimates can be also assessed by perturbing the seismic efficiency according to its fractional uncertainty in each volcano, recalculating the dike volume at the time prior to eruption, and extracting $K_c$ values from the Monte Carlo simulations as before. Table S1 lists the upper and lower values of fracture toughness derived from this procedure for each volcano, where it can be seen that both groups of values lie within the uncertainty range (${\pm }$30%) delimited in Fig. 4.

### The dynamics of propagating dikes

**Figure 5:**
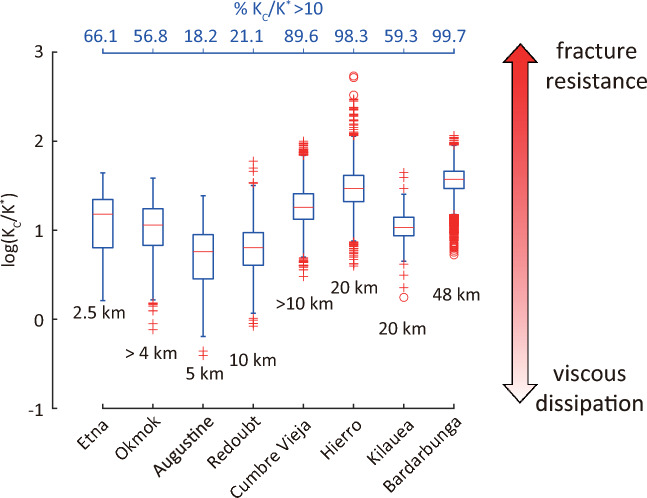
Diagram depicting the dynamical propagation regime of the eight dikes under study in the form of boxplots that summarize the statistical properties of the $K_c/K^*$ ratio distribution. Crosses denote points between 1.5 and 3 times the interquartile range, while circles are points that lie more than 3 times outside the interquartile range. The approximate length of the dikes as deduced from geophysical observations are also shown at the bottom of each boxplot (see supplementary information for details).

A simple way to investigate the dynamics of dike propagation is to consider the balance between the flow of viscous magma in the dike and the resistance of the solid medium to fracture. A measure of the contribution of viscous flow to the propagation of a dike can be given $\hbox {by}^{4,18}$

4 $$\begin{aligned} K^{*} = \Big [\frac{G^3}{(1-{\nu })^3} \frac{3{\mu }Q}{2}\Big ]^{\frac{1}{4}} \end{aligned}$$

where ${\mu }$ is magma viscosity, *Q* is flux rate of magma in the dike and all other parameters are the same as defined previously. If $K_c / K^* \gg$ 1 then resistance to fracture is the dominant process and additional bouyancy is required in order to overcome the resistance of the medium. Resistance to fracture can also be considered as the cause of prohibiting the dike from reaching large distances away from the magma chamber, and/or stopping its propagation to the surface. At the other end, if $K_c / K^* \ll$ 1 it is expected that dike growth is only limited by the viscosity of the magma that flows inside the dike (viscous dissipation regime). One difficulty in using $K_c / K^*$ for assessing dike dynamics is the fact that the flux rate *Q* is seldom known. Another problem is that *Q* is unlikely to remain constant throughout the propagation, which implies that $K_c / K^*$ may exhibit significant fluctuations that could shift the propagation regime from fracture resistance to viscous dissipation and vice versa. The reconstruction of dike volume histories presented earlier allows one to calculate *Q* from the early stages of unrest until the occurrence time of the eruptions. The magma flux rate *Q* (in $\hbox {m}^3$ $\hbox {s}^{-1}$) inside each dike can then be approximated as

5 $$\begin{aligned} Q \approx \frac{{\Delta }V(t_{i+1}) - {\Delta }V(t_{i})}{t_{i+1} - t_{i}} \end{aligned}$$

where the index $i =$ 1, 2,...,*n* represents the number of consecutive values of ${\Delta }V$ at times $t_i$. In order to be utilized for the calculation of $K_c / K^*$ ratio, the flux rate is adjusted to units of $\hbox {m}^3$ $\hbox {s}^{-1}$ $\hbox {m}^{-1}$ by taking into account the length of each dike. Magma viscosity is likely to vary significantly among the eight eruptions, since five of them (Bardarbunga, Etna, Hierro, Cumbre Vieja, Kilauea) erupted basaltic magma and the other three (Augustine, Redoubt, Okmok) erupted basaltic andesite or andesite^32–34^. Takeuchi^35^ calculated pre-eruptive magma viscosity for a variety of compositions that ranged from basaltic to rhyolitic magma. Based on these results basaltic magma containing 46–50 wt.% $\hbox {SiO}_2$ has a pre-eruptive viscosity between 100–300 Pa s, hence the median value of 200 Pa s is adopted for the five basaltic eruptions. The eruptions in the three Alaskan volcanoes involved magma containing 55–63 wt.% $\hbox {SiO}_2$ corresponding to a viscosity range of $\textrm{10}^{3.8}{-}\textrm{10}^{4.2}$ Pa s, thus a median pre-eruptive viscosity of $\textrm{10}^4$ Pa s was selected for the calculations. The rigidity modulus was set equal to 3 GPa and the Poisson ratio was taken as 0.31 which represents a value between intact and highly fractured volcanic rock^36^. The distribution of $K_c / K^*$ for each dike is presented in the form of boxplots along with the percentage of values that exceed 10, and the approximate length of each dike (Fig. 5).

The dikes at Hierro, Bardarbunga, Cumbre Vieja, Okmok, and Etna exhibit the highest percentages (56.8–99.7%) of $K_c / K^*$ above 10, thus dike propagation was mostly influenced by fracture resistance. In Hierro the dike propagated laterally for about 20 km before feeding the 2011 submarine eruption^37^. Analog experiments have shown that magma anti-buoyancy is not the only factor causing lateral dike propagation, and that rigidity layering of the upper crust also plays an important role^38^. A rigidity contrast of up to 9% between stiff and soft layers has been found to promote lateral dike propagation along the contact^39^. It is likely therefore that a rigidity contrast larger than this along the path of the Hierro dike resulted in increased fracture resistance. In Bardarbunga and Cumbre Vieja the influence of fracture resistance can be explained by considering that in both cases the dikes were segmented. Dike segmentation implies that at the end of each segment barriers had to be overcome in order for the dike to form a new segment as has been observed at Bardarbunga^27^, and could also be inferred by the twisted and bent path of the dike at Cumbre Vieja^40^. The dike during the 2008 Etna eruption also exhibits a high percentage of values above 10 with fracture resistance likely being a significant factor. Dikes in Etna usually propagate through the central conduit system, however, the dike in 2008 diverged from this path and propagated through a part of the edifice that was not previously fractured^41,42^. This probably resulted in a propagation mode that was affected by the heterogeneity of the edifice material and its resistance to fracture. In a similar way, Okmok is a caldera whose shallow structure is dominated by a mixture of lavas and pyroclastic deposits^34^ that would require more energy to fracture, thus justifying the relatively large percentage (${\sim }$56.8%) of $K_c / K^*$ above 10.

The dikes at Augustine, Redoubt, and Kilauea exhibit smaller percentages of $K_c / K^*$ above 10, pointing to a propagation regime mostly influenced by viscous dissipation. For the first two cases, this can be interpreted as a result of dike paths that followed the central conduit system, thus exploiting pre-existing zones of weakness, therefore the influence of fracture resistance was rather small. The lateral dike at Kilauea seems to be less influenced by fracture resistance compared to Hierro, even though they both exhibit similar length (${\sim }$20 km) and fracture toughness (334 vs. 386 MPa $\hbox {m}^{1/2}$). The cause for this is probably the depths at which the two dikes propagated, namely 10–18 km for Hierro^37,43^ and less than 6 km for Kilauea^44^. A dike-chamber model that was used to simulate the Kilauea dike growth indicated that rocks along its path likely exhibited rigidity that was lower than 3 GPa^45^. It is reasonable then to assume that the rigidity contrast in the different layers of the upper crust in Hawaii’s East Rift Zone was within 9% promoting an efficient lateral propagation unlike the dike at Hierro.

## Discussion

### Weertman versus lubrication model

From a theoretical point of view dike propagation is described in terms of the Weertman and lubrication models, where each of them entails different approximations and assumptions^4^. The Weertman model is considered as a suitable description of dike dynamics when the magma exhibits low viscosity, thus being able to vacate the tail of the dike. This results in the detachment of the dike from the magma source after which point the volume of magma within the dike remains constant. In this case the assumption of low viscosity for the magma also implies that dike propagation is mostly influenced by fracture resistance. The results presented earlier have provided evidence that this may be the case for the dikes at Hierro, Bardarbunga, and Cumbre Vieja that are characterized by high percentage of $K_c/K^*>$ 10 and are filled with low viscosity basaltic magma. Furthermore, their volume histories show that there was very little magma injected in each dike after the start of the eruption, which can be interpreted as a sign that the dike tail was effectively disconnected from the magma source. The lubrication model can be considered more representative of dikes filled with higher viscosity magma of which some remains in the tail, keeping it connected to the magma source. The dikes at Augustine and Redoubt seem to be better described by this model, on account of their low percentage of $K_c/K^*>$ 10 and the fact that dike volume kept increasing after eruption, hence the connection with the magma source was maintained. For the same reasons dike propagation at Kilauea can also be approximated by the lubrication model, which at first seems counterintuitive since Kilauea erupts predominantly low viscosity basaltic magma. This apparent contradiction can be explained by considering that magma in the early stages of the 2018 eruption was basaltic andesite with viscosity higher than $\textrm{10}^{2.5}$ Pa s^46^. This leaves the dikes at Okmok and Etna resembling the behavior of the Weertman model, but still being connected to the magma source as evidenced from the dike volume increase after the eruption began. All these observations thus reinforce the argument that the Weertman and lubrication models represent two extremes from the range of in-between models of how dikes propagate in volcanic environments.

### Fracture toughness and dike length

The results presented here also shed some light on the dependence of dike propagation on the material properties and the influence of dike length on fracture toughness. As mentioned earlier, volcanoes whose edifice or caldera infill consists of a heterogeneous mixture of stiff lavas and soft pyroclastic deposits are expected to exhibit larger resistance to fracture. The results presented in this work reveal that in the case of edifices this is true only when the dike path traverses a part not previously fractured, as in the case of the 2008 Etna eruption. Dikes that follow the path of the central conduit traverse the part of the edifice that has been already weakened by past intrusions (as in Augustine, Redoubt), hence resistance to fracture is small and viscous dissipation dominates the dynamics. Laboratory experiments indicate an increase of fracture toughness with the size of the process zone of cracks^4^, while numerical models^47^ suggest a power law of the form $K_c \propto L^{\alpha }$, where *L* is the length of the dike or hydrofracture and the exponent ${\alpha }$ is smaller or equal to 1/2. It is interesting to note that Scholz^10^ derived a similar power law with ${\alpha } =$ 1/2 from observations of mode I fractures such as solidified dikes, veins, and joints. The power law model of fracture toughness also predicts that beyond a certain length scale, $K_c$ should saturate, thus offering a compromise between the contrasting views of a constant (material-dependent) versus a variable fracture toughness. The number of observations in this study is too small in order to attempt deriving an empirical relationship between $K_c$ and dike length that could be used to test the validity of the aforementioned power law in the case of dynamically propagating dikes. However, an increase in fracture toughness by a factor of 6.8 (123–833 MPa $\hbox {m}^{1/2}$) can be clearly observed when the dike length increases from 2.5 to 48 km.

### Solidified versus propagating dikes

A comparison of the fracture toughness obtained here with that derived from the geometrical properties of solidified dikes (38–273 MPa $\hbox {m}^{1/2}$) shows a significant difference between the two ranges. This difference cannot be attributed to the magma composition or the length of the solidified dikes, since they covered compositions from basaltic to silicic and had lengths from few hundred meters up to 56 km^13–15^. Gill et al.^17^ developed an analytical model of how the geometrical properties of dikes change after cooling and solidification. According to this model, after substantial time has passed since the emplacement of the dike, the length starts to increase at the expense of the thickness causing the thinning (and thus volume shrinking) of the dike. The exact amount of this thinning depends on the cooling history which may vary considerably for different settings. It is likely therefore that estimates of fracture toughness from solidifed dikes represent a lower bound relative to the ones obtained here for dynamically propagating dikes.

### Limitations of dike volume calculation

The limitations of the methodology presented in this study concern the quality of earthquake catalogs, the uncertainty involved in the estimation of seismic efficiency as well as the uncertainties in the conversion from local to moment magnitudes. The first limitation requires a local seismic network that maintains a low and stable completeness magnitude over time, thus ensuring that all events that contribute significantly to seismic moment release are identified. The second limitation stems from the fact that except from seismic moment release, a geodetic estimate of intrusion volume or the total volume of erupted material is needed in order to calculate seismic efficiency. In many cases the uncertainties for either volume are relatively large, thus increasing also the uncertainty in the calculated values of seismic efficiency. However, as shown previously fractional uncertainties in seismic efficiency between 4–40% still yield fracture toughness estimates within the expected unecrtainty obtained from the Monte Carlo simulations. Finally, the third limitation has to do with the fact that the conversion relationship of $M_L$ to *Mw*, utilized here and in Kettlety et al.^20^, assumes that 3.0 is the cross-over magnitude above which $M_L$ and $M_w$ can be considered equivalent (see Methods). However, $\hbox {Deichmann}^{49}$ showed that the cross-over magnitude is likely not a constant, but rather changes according to the scattering properties of the solid medium, which means that each volcano may exhibit its own cross-over magnitude. This limitation can potentially be removed in the future by performing an empirical calibration of one magnitude scale against the other.

## Conclusions

The reconstruction of dike volume histories presented in this study can be utilized for studying dike propagation at volcanoes where geodetic observations are either not available, or the deformation signal is overprinted by the simultaneous deflation of the magma chamber. Seismic efficiency appears to vary little for each volcano irrespectively of what kind of volume (intrusion or erupted) was employed for its calculation. This means that if such volumes are known from a past eruption or intrusion, it is possible to estimate seismic efficiency and to use this value for reconstructing the volume history of any future intrusion. The methodology described here has the potential to become a useful tool for (1) monitoring the growth of dikes in real time, (2) for constraining fracture toughness in order to be used as input to analog or numerical dike propagation models, and (3) for providing volcano observatories and civil protection agencies with a quick assessment for the magma volume of an impending eruption.

## Methods

### Calculation of moment magnitudes and seismic moment

Four of the earthquake catalogs utilized in this work (Augustine, Redoubt, Okmok, Bardarbunga) contain local magnitudes ($M_L$) for all the events that fall within the spatiotemporal window selected. Local magnitudes need to be converted into moment magnitudes in order to obtain seismic moment release for each earthquake. For earthquakes with $M_L \ge$ 3.0 it is reasonable to assume the equivalence of local and moment magnitude ($M_L \approx Mw$). However, this is not possible for smaller earthquakes whose wavefield is more sensitive to the scattering properties of the medium^48^. Most of the volcanotectonic earthquakes have local magnitudes smaller than 3.0 therefore for these events conversion to *Mw* was performed by the following relationship^20,48^

6 $$\begin{aligned} Mw = \frac{2}{3} M_L + 1.0 \hspace{0.5 cm} (M_L < 3.0) \end{aligned}$$

The enhanced catalog for Kilauea contained duration magnitudes for the majority of the events and local magnitudes for the larger events. There is no empirical relationship to convert one magnitude scale to the other for $\hbox {Hawaii}^{50}$, therefore events were selected not only based on the spatiotemporal window, but also based on whether local magnitudes were available. These magnitudes were then converted to moment magnitudes as outlined above. In the supplementary information (Fig. S3) it can be seen that when using all events in the Kilauea catalog (thus treating duration magnitudes as local magnitudes) results in larger uncertainties for volume change and an overestimation of the total accumulated volume relative to the DRE erupted volume.

The routine earthquake catalogs related to the 2011 Hierro and 2021 Cumbre Vieja eruptions were obtained from Instituto Geográfico Nacional (IGN) of Spain and contained magnitudes calculated based on the amplitude of the *Lg* phase (denoted as *mbLg*). The conversion of this type of magnitude to moment magnitude was performed by using empirical relationships developed by del Fresno et al.^50^

7 $$\begin{aligned} Mw= & {} 0.66mbLg + 0.84 \hspace{0.5 cm} (mbLg < 4.0) \end{aligned}$$

8 $$\begin{aligned} Mw= & {} mbLg - 0.4 \hspace{0.5 cm} (mbLg \ge 4.0) \end{aligned}$$

The enhanced earthquake catalog for the 2021 Cumbre Vieja $\hbox {eruption}^{52}$ was merged with the IGN catalog, and already included local magnitudes that were converted into *Mw* as outlined above.

The earthquake catalog for Etna was extracted from the work of Alparone et al.^52^ that covered the period from 2000 until 2010 and contained a mixture of duration and local magnitudes with many events having both. It was then possible to use these events in order to perform an empirical calibration and obtain a relationship that would convert duration to local magnitude. Orthogonal $\hbox {regression}^{54}$ was employed for this purpose under the assumption that the ratio of the variances of uncertainties for the two magnitude scales is equal to unity (see Text S2 and Fig. S4 in suppl. information). The regression equation that was obtained is

9 $$\begin{aligned} M_L = 1.27 (\pm 0.01)M_d - 0.55 (\pm 0.19) \end{aligned}$$

where $M_d$ denotes duration magnitude and the uncertainty of each coefficient is shown in brackets. It was then possible to convert duration magnitudes of events during the 2008 eruption into local magnitudes and then into moment magnitudes as described earlier. Once magnitudes of all catalogs were converted to *Mw* seismic moment was calculated (in N m) as $M_{0} = 10^{(3/2)(Mw + 6.0)}$ and then cumulatively summed in order to obtain ${\Sigma }M_0$ for each eruption.

### Estimation of uncertainties

The calculation of seismic moment uncertainty follows the procedure used by Kettlety et al.^20^ in assigning fractional uncertainty for the calculated $M_0$ based on the magnitude of each event. According to this scheme fractional uncertainty for $M_0$ decreases linearly from 80% to 30% for events with *Mw* in the range of 0.0–4.0, which is the magnitude range observed in all catalogs. The reasoning for choosing these values as endpoints of fractional uncertainty stems from the fact that uncertainty is usually larger for smaller ($M<$ 2) events and a fractional uncertainty up to 80% is expected, as shown in previous $\hbox {studies}^{55}$. The uncertainty of the cumulative seismic moment release is then obtained by summing in quadrature the fractional uncertainties of each event. The uncertainty of seismic efficiency was estimated using error propagation by taking into account the fractional uncertainty of eruption volume ${\delta }V/V$ and the fractional uncertainty of the summed seismic moment ${\delta }{\Sigma }M_{0}/{\Sigma }M_{0}$ (see Table 1 and the supplementary information).

### Monte Carlo simulation

The calculation of critical volume requires, except from dike properties, knowledge of the Poisson ratio and shear modulus along the path of each dike under study. Since this knowledge is in most cases not available, a Monte Carlo framework was employed in order to simulate critical volume within a range of these parameters and then consider the statistical properties of the resulting distributions. Heap et al.^36^ have suggested realistic values of elastic moduli that can be used in such calculations, based on laboratory and field studies. The authors concluded that Poisson ratio in volcanic rocks range from 0.21 for intact rocks to 0.41 for highly fractured ones, while Young’s modulus *E* ranges between 1 and 15 GPa. Having this information available it is possible to calculate the shear modulus as

10 $$\begin{aligned} G = \frac{E}{2(1+ {\nu })} \end{aligned}$$

Based on a Monte Carlo simulation sampling $E, {\nu }$ from the aforementioned ranges, the median value of shear modulus was found to be close to 3 GPa. The calculation of $V_c$ also requires values such as fracture toughness $K_c$, density difference ${\Delta }{\rho }$, and propagation angle ${\theta }$ of the dike. A wide range of fracture toughness was selected ranging from 10 MPa $\hbox {m}^{1/2}$, close to reported laboratory measurements, up to 2000 MPa $\hbox {m}^{1/2}$. The range for the density difference starts at 20 kg $\hbox {m}^{-3}$, which implies anti-buoyant behavior for the magma, up to a value of 250 kg $\hbox {m}^{-3}$ for fully buoyant magma. The angle away from the vertical was allowed to range from zero degrees (vertical dike) to $85^{\circ }$ which is consistent with a laterally propagating dike. A uniformly sampled but random combination of these parameters was used in order to calculate the critical volume for 1.5 million times.

### Supplementary Information

Supplementary Information.

## Data Availability

The routine catalogs for Hierro and Cumbre Vieja can be downloaded directly from the IGN website (https://www.ign.es/web/en/ign/portal/sis-catalogo-terremotos). The routine catalog for the three Alaskan volcanoes (Augustine, Redoubt, Okmok) is publicly available as part of the electronic supplement of Power et al.^55^. Information for the end time of each eruption was obtained from the Global Volcanism Database of the Smithsonian Institution (https://volcano.si.edu/search_volcano.cfm). All other catalogs can be found in the papers cited in the manuscript and/or supplementary material.
