# Supplementary material for: Dike volume derived from seismicity as a gauge of fracture toughness and propagation dynamics
Source: Sci Rep. 2024 Jul 30;14:17593. doi: 10.1038/s41598-024-67724-0 (PMC11289457; doi:10.1038/s41598-024-67724-0)
Supplement: Supplementary file 1 — Supplementary Information. [file 41598_2024_67724_MOESM1_ESM.pdf]

Supplementary information for

Dike volume derived from seismicity as a gauge of fracture toughness and propagation dynamics

K. I. Konstantinou

Dept of Earth Sciences, National Central University, Jhongli, 320 Taiwan
Email: kkonst@ncu.edu.tw Fax: ++886-3-4222044

Content description

Text S1: Description of earthquake catalogs, spatiotemporal windows, DRE volumes, dike length

Text S2: Empirical calibration of local and duration magnitudes at Etna

Table S1: Sensitivity of fracture toughness to perturbation of seismic efficiency.

Figure S1: Frequency-magnitude distributions and marked completeness magnitudes for each catalog.

Figure S2: Normalized histograms of fracture toughness inferred from geodetically determined dike volume for Bardarbunga, Kilauea, and Okmok volcanoes.

Figure S3: Dike volume history for Kilauea when all magnitudes (duration and local) are used.

Figure S4: Results of orthogonal regression for duration and local magnitudes in Etna.

Text S1

Augustine (2006)

The Alaska Volcano Observatory (AVO) catalog provided by Power et al. (2019) was utilized for extracting events within the spatiotemporal window defined below. The seismicity rate started increasing in early May 2005 along with deformation (Cervelli et al., 2006; Jakobs and McNutt, 2010), while the flux of gas derived from the magma reservoir increased significantly by August 2005 (Zhan et al., 2022). The start date of the window was set 2 May 2005 since this was the date of the first event that month. A radius of 2 km (see Buurman et al., 2014) centered at the peak of the edifice and down to a depth of 6 km was used as the spatial filter. The total Dense Rock Equivalent (DRE) erupted volume was estimated to be $72.7(\pm 8.0)\times 10^6$ m^3 (Coombs et al., 2010). The dike length is constrained to about 5 km based on the vertical extent of the seismicity and petrological data (Coombs et al., 2010; Zhan et al., 2022).

Etna (2008)

The Etna earthquake catalog of Alparone et al. (2015) was utilized for extracting events within the spatiotemporal window defined below. An increase in seismicity both in event number and magnitudes as well as deformation was observed in early January 2008 (Alparone et al., 2012), hence 1 January 2008 was set as the start date of the window. In terms of spatial extent, the catalog already covered the main edifice of Etna within a radius of 10 km around the peak and a depth down to 6 km was covered by the majority of the events during the period considered. The bulk erupted lava volume of the 2008 eruption was estimated from the comparison of digital elevation models obtained before and after eruptive activity and was found to be $79.7(\pm 3.61)\times 10^6$ m^3 (Behnke et al., 2016). The dike length was constrained to be 2.5 km based on joint modeling of magnetic, gravity and deformation observations (Aloisi et al., 2009; Currenti et al., 2011).

Okmok (2008)

The AVO catalog was utilized for extracting events within the spatiotemporal window defined below. GPS observations indicated that recharge of the magma reservoir started almost immediately

after the 1997 eruption, however, the bulk of the deformation started occurring after January 2003 (Fournier et al., 2009; Lu et al., 2010; Biggs et al., 2010). Continuous seismic monitoring started in early 2003 (Ohlendorf et al., 2014), therefore the starting date of the window was set to 17 January 2003 when the first event at the volcano was detected. Garza-Girón et al. (2023) compiled an enhanced catalog by use of waveform template matching for the period between 2 June until 31 August 2008. The two catalogs were merged taking care to exclude common events. A radius of 10 km (see Buurman et al., 2014) around the center of the caldera and down to a depth of about 6 km was used as the spatial filter. The DRE erupted volume was estimated to be $0.21(\pm 0.05)\times 10^9$ m^3 (Larsen et al., 2015). The dike length is constrained between 4-6 km based on the vertical extent of the seismicity and deformation data (Ohlendorf et al., 2014; Garza-Girón et al., 2023; Fournier et al., 2009; Lu et al., 2010; Biggs et al., 2010).

Redoubt (2009)

The AVO catalog was utilized for extracting events within the spatiotemporal window defined below. Anomalous ground deformation was detected already in early May 2008 (Grapethin et al., 2013), followed by increased sulfur odor in mid-July, and snow melt in mid-September (Power et al., 2013). However, volcanotectonic seismicity did not start until 1 October 2008 which was chosen as the starting date of the window. A radius of 8 km (see Buurman et al., 2014) centered at the peak of the edifice and down to a depth of 10 km was used as the spatial filter. The DRE erupted volume was estimated to $10.0(\pm 5.0)\times 10^7$ m^3 (Power et al., 2013). The dike length can be constrained to about 10 km using the seismicity distribution and petrological data (Bull and Buurman, 2013; Power et al., 2013).

Hierro (2011)

The routine catalog of Instituto Geográfico Nacional (IGN) of Spain was utilized for extracting events within the spatiotemporal window defined below. Seismicity started in early July 2011 along with deformation in the north of El Hierro island and progressively migrated to the south by early September 2011 (López et al., 2012). The start date of the window was therefore set 20 July 2011 since the seismicity increased significantly after that date. The spatial extent of the window was

defined by the distribution of seismicity in published studies (such as López et al., 2012) and the depth was restricted to the range between 10-18 km. A large event (Mw ∼4) occurred on 8 October 2011 near the southern tip of the island, however, its moment tensor did not exhibit any non-double couple components, hence it was most likely a shear failure event not related to the propagating dike (López et al., 2017). This event along with its aftershocks were excluded from the selected event catalog. The DRE erupted volume was estimated to $20(\pm 8)\times 10^7$ m^3 (Martí et al., 2013). It should be noted that the erupted volume uncertainty was not given by Martí et al. therefore it was assumed to be 40% of the reported volume. This rather large uncertainty was adopted as a worst-case scenario and the true uncertainty is likely smaller. The dike length can be constrained to about 20 km based on the horizontal extent of the relocated seismicity (Dóminguez-Cerdeña et al., 2014).

Bardarbunga (2014)

The catalog provided by Ágústsdóttir et al. (2019) was utilized for extracting events within the spatiotemporal window defined below. The seismic swarm that was related to the dike formation started on 16 August 2014, hence this was set as the start date of the window. The spatial window follows Kettlety et al. (2022) in the sense that events were excluded if they were unrelated to the dike growth (i.e events related to the caldera collapse). The DRE erupted volume of the Bardarbunga-Holuhraun eruption was estimated using satellite and ground-based measurements to be $1.21(\pm 0.10)\times 10^9$ m^3 (Bonny et al., 2018). The dike length has been estimated using different datasets: GPS observations ∼45 km (Sigmundsson et al., 2015); seismicity and deformation ∼48 km (Gudmundsson et a., 2016); detailed mapping of seismicity distribution ∼50 km (Woods et al., 2019). A median value of 48 km was adopted here.

Kilauea (2018)

The enhanced catalog of Wei et al. (2022) was utilized for extracting events within the spatiotemporal window defined below. This catalog was selected because it covers the period of the Kilauea eruption and uses advanced techniques of event detection and phase association applied to waveforms recorded by all available stations, both onshore and offshore (i.e OBS). The first signs (in

the form of deformation and seismicity) that magma was migrating east of the Pu'u O'o crater into the lower East Rift Zone, were detected on 30 April 2018, hence this was set as the start date of the window. The spatial window takes into account the fact that on 4 May a large (Mw ~7.2) earthquake occurred in the offshore south flank of Kilauea, probably triggered by the dike intrusion. This event along with its aftershocks were excluded from the catalog, based on its slip distribution published by Chen et al. (2019). All other events east of the Pu'u O'o crater and up to the Leilani Estates were utilized in this work. As mentioned in the main text only the large events had local magnitudes, while smaller events (M $\leq$ 1.4) had only duration magnitudes available. There is no empirical relationship to convert the one scale into the other for Hawaii, and if all events are used for estimating the dike volume history then an overestimation will occur (Figure S3). However, this does not constitute a problem since the small events contribute very little to the cumulative seismic moment, hence they can be excluded in favor of the larger events with local magnitudes. The DRE erupted volume of the Kilauea eruption was estimated using a variety of techniques (eruption videos, lidar, DEMs etc) to be $1.15(\pm 0.25)\times 10^9$ m^3 (Dietterich et al., 2021). The dike length was estimated to be ~20 km long, based on geodetic and seismic observations (Chen et al., 2019; Legliné et al., 2021).

Cumbre Vieja (2021)

The routine catalog of IGN was utilized for extracting events within the spatiotemporal window defined below. Deformation derived from DInSAR observations and precursory seismicity started occurring between 8-16 September 2021 (De Luca et al., 2022; D'Auria et al., 2022), hence 11 September was set as the start date of the window. The spatial window was determined by considering only the seismicity that was surrounding the deformation anomaly prior to eruption and the location of the vent that opened after the dike reached the surface. Only events with hypocentral depths of 10 km or shallower were included, since deeper events represented changes at the deeper plumbing system of the volcano (D'Auria et al., 2022; del Fresno et al., 2023). Suarez et al. (2023) compiled an enhanced catalog of the seismicity by use of deep learning for event detection and phase assosiation that covered the period from 11 until 19 September. This catalog was merged with the IGN catalog after excluding common events and applying the same spatial filter. The bulk erupted lava volume of the eruption using thermal and optical satellite

observations was estimated to be $212(\pm 13)\times 10^6$ m^3 (Plank et al., 2023). The dike length was estimated to be $\sim$10 km based on the seismicity distribution, however, the fact that the dike bent in order to deflect away from the volcano edifice (Przeor et al., 2024) suggests that this estimate is likely a lower bound.

Text S2

Castellaro et al. (2006) were the first to point out that regression of two magnitude scales entails uncertainties in both kinds of magnitude, which the standard least-squares regression does not take into account. Instead the authors advocated the use of General Orthogonal Regression (hereafter referred to as GOR). GOR takes into account uncertainties in both variables by considering the ratio of their variances $\eta = \sigma_y^2/\sigma_x^2$ and fits the line by minimizing the Euclidean distance between the points and the line (in the special case where $\eta = 1$ this distance corresponds to the orthogonal distance). In practice the application of GOR to real magnitude data hinges upon the fact that in most cases the value of the variance ratio is not known for the reason that the individual variances (σ_y^2, σ_x^2) are also unknown. In this case Castellaro and Bormann (2007) propose that the regression can be carried out by assuming that $\eta = 1$ where GOR becomes Orthogonal Regression (hereafter referred to as OR). The catalog of Alparone et al. (2015) for seismicity at Etna contains 2523 events that have both duration and local magnitude, however, the catalog does not contain information about the uncertainties of either magnitude scale. Therefore it was assumed that the uncertainty variance of both magnitude scales is the same, in which case $\eta = 1$ and OR can be applied. This assumption is reasonable by considering the fact that the different magnitude estimates have been obtained by using similar approaches, in the sense that a single measurement (S-wave amplitude or signal duration) is used in order to calculate the magnitude utilizing an empirical relationship. Figure S4 shows a plot of the pairs of points and the regressed line along with its 95% confidence intervals.

References

Ágústsdóttir T., T. Winder, J. Woods, R. S. White, T. Greenfield, B. Brandsdóttir, 2019. Intense seismicity during the 2014-2015 Bardarbunga-Holuhraun rifting event, Iceland, reveals the

nature of dike-induced earthquakes and caldera collapse mechanisms, J. Geophys. Res. Solid Earth, 124, 8331-8357, https://doi.org/10.1029/2018JB016010

Aloisi M., A. Bonaccorso, F. Cannavó, S. Gambino, M. Mattia, G. Puglisi, E. Boschi, 2009. A new dyke intrusion style for the Mount Etna may 2008 eruption modelled through continuous tilt and GPS data, Terra Nova, 21, 316-321, https://doi.org/10.1111/j.1365-3121.2009.00889.x

Alparone S., G. Barberi, O. Cocina, E. Giampiccolo, C. Musumeci, D. Patané, 2012. Intrusive mechanism of the 2008-2009 Mt Etna eruption: Constraints by tomographic images and stress tensor analysis, J. Volc. Geotherm. Res., 229-230, 50-63, https://doi.org/10.1016/j.volgeores.2012.04.001

Alparone S. et al., 2015. Instrumental catalog of Mt Etna earthquakes (Sicily, Italy): ten years (2000-2010) of instrumental recordings, Ann. Geophys., 58, S0435, https://doi.org/10.4401/ag-6591

Behnke B., A. Fornaciai, M. Neri, M. Favalli, G. Ganci, F. Mazzarini, 2016. Lidar surveys reveal eruptive volumes and rates at Etna, 2007-2010, Geophys. Res. Lett., 43, 4270-4278, https://doi.org/10.1029/2016GL068495

Biggs J., Z. Lu, T. Fournier, J. T. Freymueller, 2010. Magma flux at Okmok volcano, Alaska, from a joint inversion of continuous GPS, campaign GPS, and inteferometric synthetic aperture radar, J. Geophys. Res., 115, B12401, https://doi.org/10.1029/2010JB007577

Bonny E., T. Thordarson, R. Wright, A. Höskuldsson, I. Jónsdóttir, 2018. The volume of lava erupted during the 2014 to 2015 eruption at Holuhraun, Iceland: A comparison between satellite- and ground-based measurements, J. Geophys. Res. Solid Earth, 123, 5412-5426, https://doi.org/10.1029/2017JB015008

Bull K. F., H. Buurman, 2013. An overview of the 2009 eruption of Redoubt volcano, Alaska, J. Volc. Geotherm. Res., 259, 2-15, https://doi.org/10.1016/j.jvolgeores.2012.06.024

Buurman H., C. J. Nye, M. E. West, C. Cameron, 2014. Regional controls on volcano seismicity along the Aleutian arc, Geophys. Geochem. Geosyst., 15, 1147-1163, https://doi.org/10.1002/2013GC005101

Castellaro S., F. Mulargia, Y. Y. Kagan, 2006. Regression problems for magnitudes, Geophys. J. Int., 165, 913-930, https://doi.org/10.1111/j.1365-246X.2006.02995.x

Castellaro S., P. Bormann, 2007. Performance of different regression procedure on the magnitude conversion problem, Bull. Seism. Soc. Am., 97, 1167-1175, https://doi.org/10.1785/0120060102

Cervelli P. F., T. Fournier, J. Freymueller, J. A. Power, 2006. Ground deformation associated with the precursory unrest and early phases of the January 2006 eruption of Augustine volcano, Alaska, Geophys. Res. Lett., 33, L18304, https://doi.org/10.1029/2006GL027219

Chen K., J. D. Smith, J.-P. Avouac, Z. Liu, Y. T. Song, A. Gualandi, 2019. Triggering of the Mw 7.2 Hawaii earthquake of 4 May 2018 by a dike intrusion, Geophys. Res. Lett., 46, 2503-2510, https://doi.org/10.1029/2018GL081428

Coombs M. L., K. F. Bull, J. W. Wallace, D. J. Schneider, E. E. Thoms, R. L. Wessels, R. G. McGinsey, 2010. Timing, Distribution, and Volume of Proximal Products of the 2006 Eruption of Augustine Volcano, In: The 2006 eruption of Augustine volcano, Alaska, J. A. Power, M. L. Coombs, J. T. Freymueller (Eds), USGS professional paper 1769

Currenti G., R. Napoli, A. Di Stefano, F. Greco, C. Del Negro, 2011. 3D integrated geophysical modeling for the 2008 magma intrusion at Etna: Constraints on rheology and dike overpressure, Phys. Earth Planet. In., 185, 44-52, https://doi.org/10.1016/j.pepi.2011.01.002

D'Auria L. et al., 2022. Rapid magma ascent beneath La Palma revealed by seismic tomography, Sci. Rep., 12:17654, https://doi.org/10.1038/s41598-022-21818-9

De Luca C., E. Valerio, F. Giudicepietro, G. Macedonio, F. Casu, R. Lanari, 2022. Pre- and co-eruptive analysis of the September 2021 eruption at Cumbre Vieja volcano (La Palma, canary islands) through DInSAR measurements and analytical modeling, Geophys. Res. Lett., 49, e2021GL097293, https://doi.org/10.1029/2021GL097293

del Fresno C. et al., 2023. Magmatic plumbing and dynamic evolution of the 2021 La Palma eruption, Nature Comms, 14:358, https://doi.org/10.1038/s4167-023-35953-y

Dietterich H. R., A. K. Diefenbach, S. A. Soule, M. H. Zoeller, M. P. Patrick, J. J. Major, P. R. Lundgren, 2021. Lava effusion rate evolution and erupted volume during the 2018 Kilauea

lower East Rift Zone eruption, Bull. Volcanol., 83:25, https://doi.org/10.1007/s00445-021-01443-6

Domínguez-Cerdeña I., C. del Fresno, A. Gomis Moreno, 2014. Seismcity patterns prior to the 2011 El Hierro eruption, Bull. Seism. Soc. Am., 104, 567-575, https://doi.org/10.1785/0120130200

Fournier T., J. Freymueller, P. Cervelli, 2009. Tracking magma volume recovery at Okmok volcano using GPS and an unscented Kalman filter, J. Geophys. Res., 114, B02405, https://doi.org/10.1029/2008JB005837

Garza-Girón R., E. M. Brodsky, Z. J. Spica, M. M. Haney, P. W. Webley, 2023. Earthquakes record cycles of opening and closing in the enhanced seismic catalog of the 2008 Okmok volcano, Alaska, eruption, J. Geophys. Res. Solid Earth, 128, e2023JB026893, https://doi.org/10.1029/2023JB026893

Grapethin R., J. Freymueller, A. M. Kaufman, 2013. Geodetic observation during the 2009 eruption at Redoubt volcano, J. Volc. Geotherm. Res., 259, 115-132

Gudmundsson M. T. et al., 2016. Gradual caldera collapse at Bardarbunga volcano, Iceland, regulated by lateral magma outflow, Science, 353, aaf8988, https://doi.org/10.1126/science.aaf8988

Jakobs K. M., S. R. McNutt, 2010. Using seismic b-values to interpret seismicity rates and physical processes during the preeruptive earthquake swarm at Augustine volcano 2005-2006, In: The 2006 eruption of Augustine volcano, Alaska, J. A. Power, M. L. Coombs, J. T. Freymueller (Eds), USGS professional paper 1769

Kettlety T., J. M. Kendall, D. C. Roman, 2022. Self-similarity of seismic moment release to volume change scaling for volcanoes: A comparison with injection-induced seismicity, Geophys. Res. Lett., 49, e2022GL099369, https://doi.org/10.1029/2022GL099369

Larsen J. F., C. A. Neal, J. R. Schaefer, A. M. Kaufman, Z. Lu, 2015. The 2008 phreatomagmatic eruption of Okmok volcano, Aleutian islands, Alaska: Chronology, deposits, and landform changes, Report of Investigations 2015-2, State of Alaska, Department of natural resources.

Lengliné O., Z. Duputel, P. G. Okubo, 2021. Tracking dike propagation leading to the 2018 Kilauea eruption, Earth Planet. Sci. Lett., 553, 116653, https://doi.org/10.1016/j.epsl.2020.116653

López C. et al., 2012. Monitoring the volcanic unrest of El Hierro (Canary islands) before the onset of the 2011-2012 submarine eruption, Geophys. Res. Lett., 39, L13303, https://doi.org/10.1029/2012GL051846

López C., M. Benito-Saz, J. Martí, C. del Fresno, L. Garcia-Cañada, H. Albert, H. Lamolda, 2017. Driving magma to the surface: The 2011-2012 El Hierro volcanic eruption, Geochem. Geophys. Geosys., 18, 3165-3184,https://doi.org/10.1002/2017GC007023

Lu Z., D. Dzurisin, J. Biggs, C. Wicks Jr, S. McNutt, 2010. Ground surface deformation patterns, magma supply, and magma storage at Okmok volcano, Alaska, from InSAR analysis: 1. Intereruption deformation, 1997-2008, J. Geophys. Res., 115, B00B02, https://doi.org/10.1029/2009JB0066969

Martí J., V. Pinel, C. López, A. geyer, R. Abella, M. Tárraga, M. J. Blanco, A. Castro, C. Rodríguez, 2013. Causes and mechanisms of the 2011-2012 El Hierro (Canary islands) submarine eruption, J. Geophys. Res., 118, 823-839, https://doi.org/10.1002/jgrb.50087

Neal C. A. et al., 2019. The 2018 rift eruption and summit collapse of Kilauea volcano, Science 363, 367-374

Ohlendorf S., C. H. Thurber, J. D. Pesicek, S. G. Prejean, 2014. Seismicity and seismic structure at Okmok volcano, Alaska, J. Volc. Geotherm. Res., 278-279, 103-119, https://doi.org/10.1016/j.volgeores.2014.04.002

Plank S. et al., 2023. Combining thermal, tri-stereo optical and bi-static InSAR satellite imagery for lava volume estimates: the 2021 Cumbre Vieja eruption, La Palma, Sci. Rep., 13:2057, https://doi.org/10.1038/s41598-023-29061-6

Power J. A., S. D. Stihler, B. A. Chouet, M. M. Haney, D. M. Ketner, 2013. Seismic observations of Redoubt Volcano, Alaska - 1989-2010 and a conceptual model of the Redoubt magmatic system, J. Volc. Geotherm. Res., 259, 31-44, https://doi.org/10.1016/j.volgeores.2012.09.014

Power J. A., P. A. Friberg, M. M. Haney, T. Parker, S. D. Stihler, J. P. Dixon, 2019. A unified catalog of earthquake hypocenters and magnitudes at volcanoes in Alaska - 1989 to 2018, USGS Scientific Investigations Report 2019-5037, 17p, doi:10.3133/sir20195037

Przeor M. et al., 2024. Geodetic imaging of magma ascent through a bent and twisted dike during the Tajogaite eruption of 2021 (La Palma, Canary islands), Sci. Rep., 14:212, https://doi.org/10.1038/s41598-023-50982-9

Sigmundsson F. et al., 2015. Segmented lateral dyke growth in a rifting event at Bardarbunga volcanic system, Iceland, Science, 517, 191-195

Suarez E. D., I. Domínguez-Cerdeña, A. Villaseñor, S. Sainz-Maza Aparicio, C. del Fresno, L. García-Cañada, 2023. Unveiling the pre-eruptive seismic series of the La Palma 2021 eruption: Insights through fully automated analysis, J. Volc. Geotherm. Res., 444, 107946, https://doi.org/10.1016/j.volgeores.2023.107946

Wei X., Y. Shen, J. Caplan-Auerbach, J. K. Morgan, 2022. An improved earthquake catalog during the 2018 Kilauea eruption from combined onshore and offshore seismic arrays, Earth Space Sci., 9, e2021EA001979, https://doi.org/10.1029/2021EA001979

Woods J., T. Winder, R. S. White, B. Brandsdóttir, 2019. Evolution of a lateral dike intrusion revealed by relatively-relocated dike-induced earthquakes: The 2014-15 Bardarbunga-Holuhraun rifting event, Iceland, Earth Planet. Sci. Lett., 506, 53-63, https://doi.org/10.1016/j.epsl.2018.10.032

Zhan Y., H. Le Mével, D. C. Roman, T. Girona, P. M. Gregg, 2022. Modeling deformation, seismicity, and thermal anomalies driven by degassing during the 2005-2006 pre-eruptive unrest of Augustine volcano, Alaska, Earth Planet. Sci. Lett., 585, 117524, https://doi.org/10.1016/j.epsl.2022.117524

Table S1. Sensitivity of fracture toughness estimates when seismic efficiency is perturbed by an amount proportional to its fractional uncertainty.

Volcano	ϵ	ΔV^+ (m^3)	K_c^+ (MPa m$^{1/2}$)	ΔV^- (m^3)	K_c^- (MPa m$^{1/2}$)
Augustine	0.11	1.48e07	176	1.86e07	194
Etna	0.04	9.40e06	137	1.03e07	140
Okmok	0.23	8.23e06	144	1.34e07	176
Redoubt	0.20	9.98e06	122	1.50e07	176
Hierro	0.40	1.15e08	304	2.72e08	475
Bardarbunga	0.13	7.99e08	725	1.05e09	868
Kilauea	0.21	6.41e07	309	9.99e07	322
Cumbre-Vieja	0.06	1.01e08	327	1.15e08	330

The quantity ϵ is equal to the fractional uncertainty of seismic efficiency ($\delta S_{eff}/S_{eff}$) at each volcano. ΔV^+ represents the volume change that acummulated prior to eruption calculated for $S_{eff} + \epsilon * S_{eff}$ yielding K_c^+ as an estimate of fracture toughness. ΔV^- represents the volume change that acummulated prior to eruption calculated for $S_{eff} - \epsilon * S_{eff}$ yielding K_c^- as an estimate of fracture toughness. In all cases the fracture toughness estimates calculated for upper or lower values of seismic efficiency are within the uncertainty range given in Figure 4 of the main manuscript.

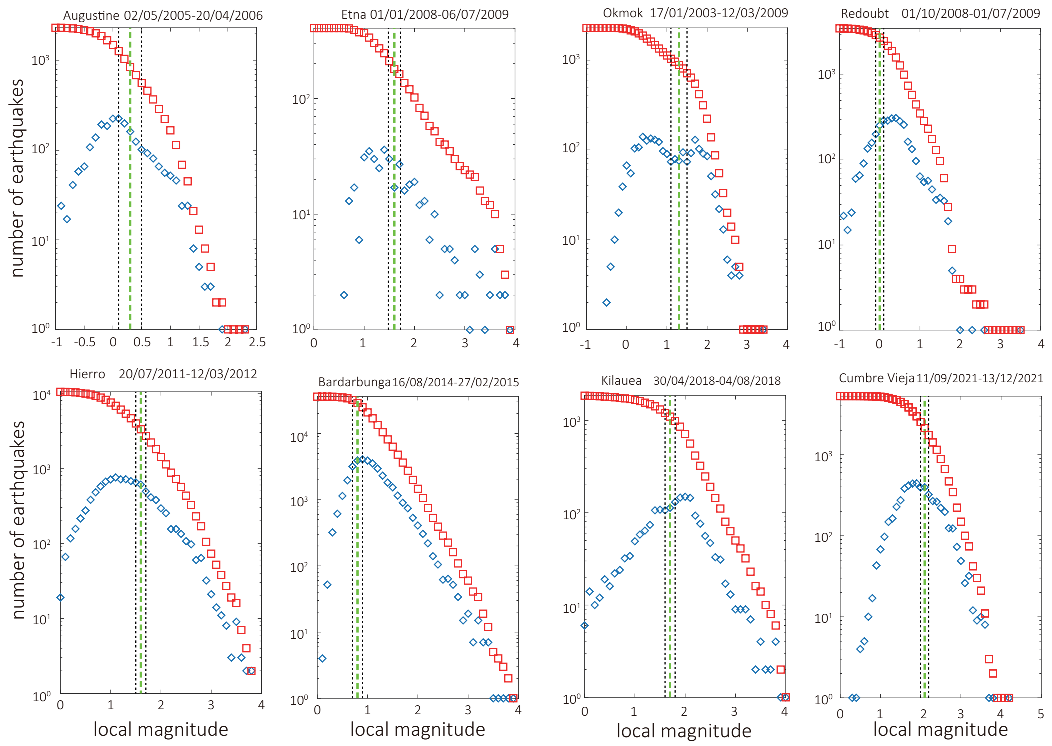

Figure S1. Frequency-magnitude distribution derived from each of the eight catalogs after event selection and conversion to local magnitudes. The red squares represent the cumulative number of earthquakes, while the blue diamonds depict the non-cumulative one. The green dashed line indicates the magnitude of completeness for each catalog estimated by using the b-value stability method. The dotted lines on either side give the uncertainty of the completeness magnitude estimated using 500 bootstrap samples in each case.

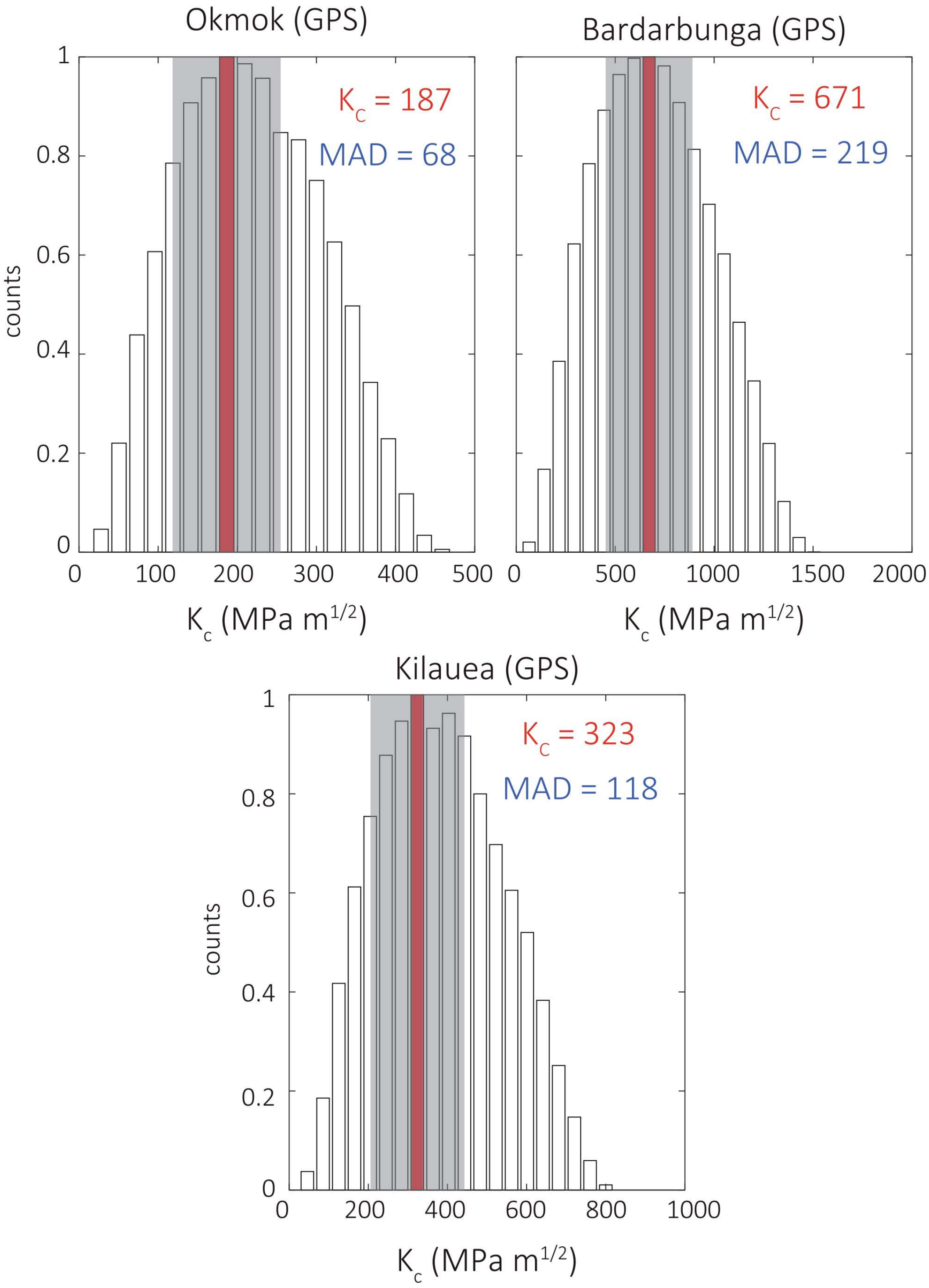

Figure S2. Normalized histograms of fracture toughness that correspond to dike volumes obtained from GPS observations that are equal to 0.75-1.0 times the critical volume. The red bar highlights the mode of each distribution. The shaded area shows the extent of the Median Absolute Deviation (MAD) on either side of the mode. The values of the mode and MAD (in MPa $m^{1/2}$) for each distribution are included at the top right corner of each plot.

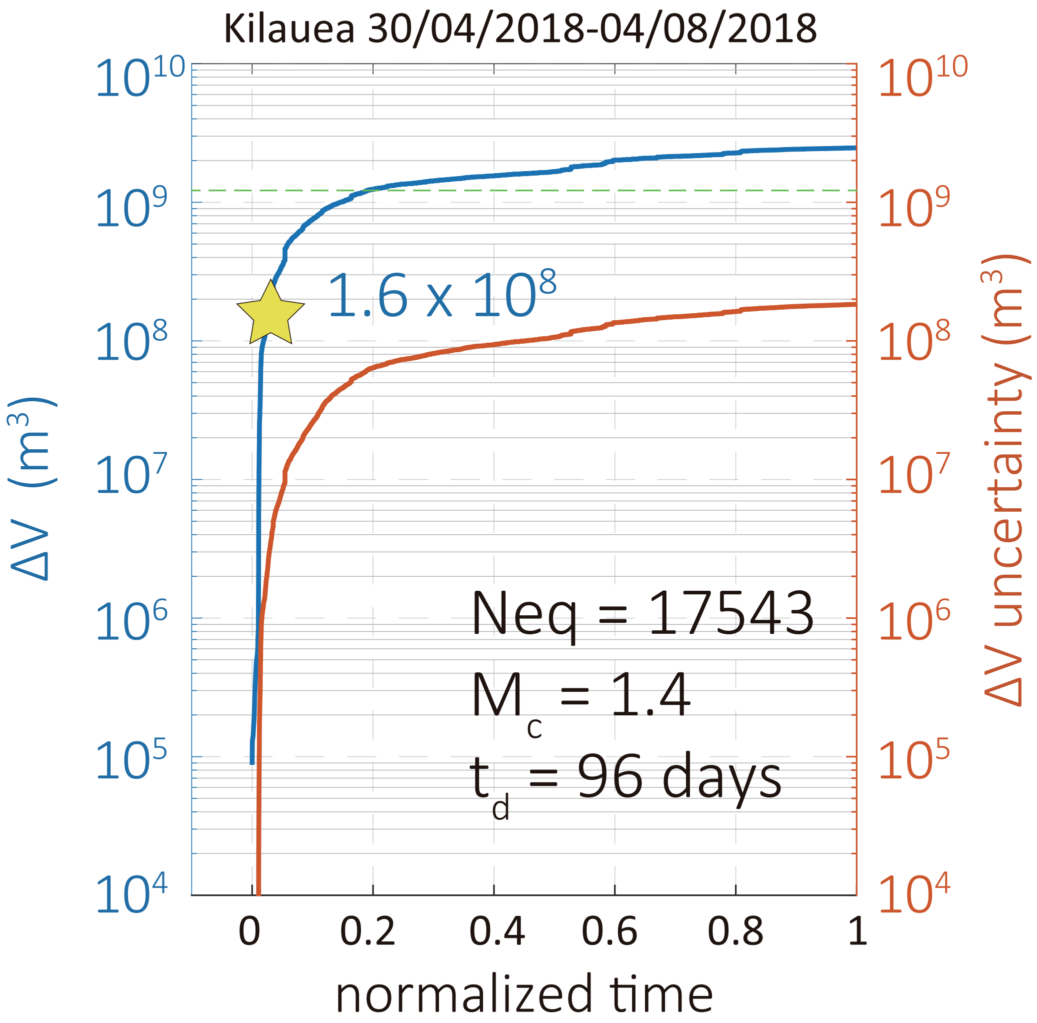

Figure S3. Dike volume history and its uncertainty calculated for the Kilauea dike if all events (with duration and local magnitudes) are included. The yellow star denotes the time of the eruption and the number in blue fonts indicates the volume (in m^3) that accumulated in the dike up to that time. Neq is the total number of volcanotectonic earthquakes included; M_c is the magnitude of completeness; t_d is the total duration of the time window which was used in order to normalize the time. The green dotted line highlights the total DRE erupted volume, which in this case has been exceeded by a factor of more than 2. A 10-fold increase in volume uncertainty can also be seen compared to the uncertainty when only local magnitudes were used for the calculation. The volume at the time of the eruption is now larger than the volume obtained from GPS (1.6×10^8 versus 10^8 m^3).

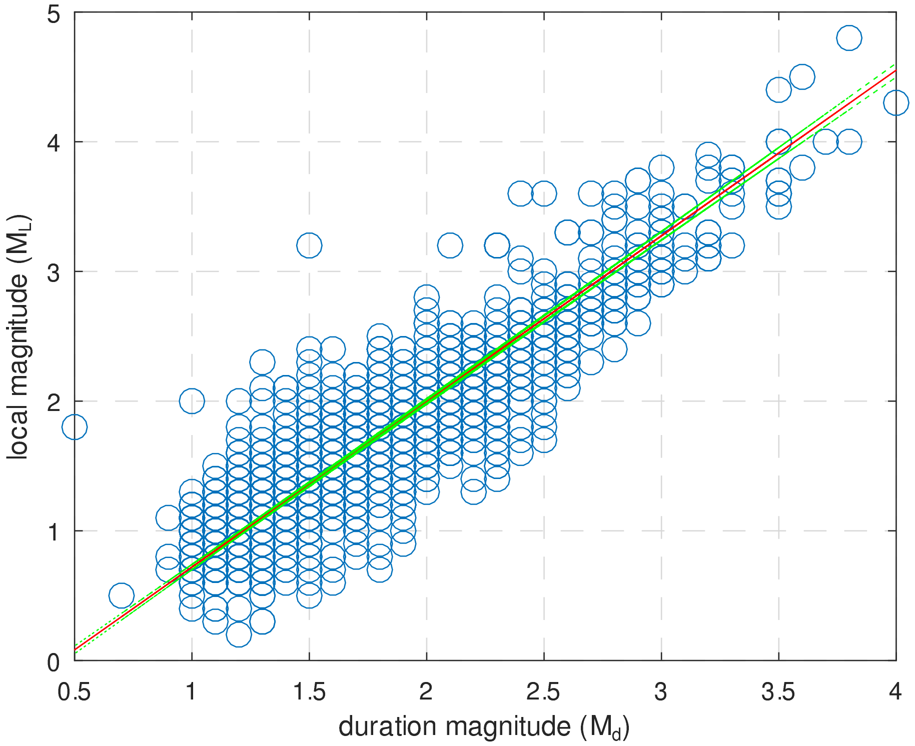

Figure S4. Plot showing the orthogonal regression results for duration and local magnitudes of 2523 events of the Etna catalog (Alparone et al., 2015). The red line is the regression line obtained and the green curves are the 95% confidence limits.
